# Commentary: The oral microbiome in young women at different stages of periodontitis: Prevotella dominant in stage III periodontitis

**DOI:** 10.3389/fcimb.2023.1141285

**Published:** 2023-02-03

**Authors:** Weiqiang Huang, Xinyu Tang, Jianfeng Bao

**Affiliations:** ^1^ The Fourth Affiliated College Of Zhejiang Chinese Medical University, Hangzhou, China; ^2^ Department of Hepatology, Affiliated Hangzhou Xixi Hospital, Zhejiang University School of Medicine, Hangzhou, Zhejiang, China

**Keywords:** periodontitis, 16S rRNA, *Prevotella*, *Porphyromonas*, systemic diseases

A study entitled “*The oral microbiome in young women at different stages of periodontitis: Prevotella dominant in stage III periodontitis* “ was recently published in Frontiers in Cellular and Infection Microbiology by Zhao et al. (2022). This study identified bacteria of the genus *Prevotella* as biomarkers of young women with stage-III periodontitis by 16S rRNA-sequencing (Zhao et al., 2022). We greatly appreciate this work and believe that the conclusion has potential value. However, we need to point out that the study protocol has some limitations, which may affect the accuracy of the conclusions.

First, only 26 young women with periodontitis were included in this study. We think that the small sample size will affect the ability to analyze alpha diversity analysis for the oral microbiome. Second, dysbiosis of oral microbial ecology is both a major causative factor for oral diseases such as periodontitis and is also closely associated with systemic diseases (Issrani et al., 2022; Xu et al., 2022). Therefore, Zhao and her colleagues excluded the group with systemic diseases, however they did not specify the methodology used to exclude systemic disease participants and did not perform detailed laboratory examinations on the individuals. It is well known that the range of systemic diseases is very broad (Bhuyan et al., 2022), and some of them have insidious clinical symptoms and are often overlooked by the patients themselves and their physicians. For example, the oral cavity and the intestine, two of the most complex microbial habitats in the body, are always interacting with each other. Numerous studies have shown that a variety of gastrointestinal diseases can alter the oral microbiota (Park et al., 2021; Abdelbary et al., 2022). For example, in *H. pylori*-positive patients, the oral microbiota is richer and more diverse than in *H. pylori*-negative patients, where *Prevotella* can reach 8.4%, second only to *Streptococcus* in terms of oral microbial abundance. In addition, viral infections such as HIV (Annavajhala et al., 2020) and HBV (Ling et al., 2015), metabolic diseases such as type 2 diabetes mellitus (Hajishengallis and Chavakis, 2021), metabolic dysfunction-associated fatty liver disease (Zeybel et al., 2022), and immune diseases such as rheumatoid arthritis (Kroese et al., 2021) can cause alterations in the composition of the host oral microbiota. Therefore, more detailed exclusion methods need to be given in the protocol, and the corresponding laboratory indicators need to be further refined for supporting evidence.

In addition, Zhao and her colleagues identified oral bacteria by amplification and sequencing of the V3-V4 region of 16S rDNA. Due to the possible limitations of this technology, sequencing the V3-V4 region of 16S rRNA can overlook species of tiny colonies and cannot reach taxonomic resolution of sparse bacterial populations at the species and strain levels. As a result, we recommend that sequencing analyses examining connections between oral microbiota and periodontitis stages should use full-length 16S intragenomic copy variations based on the V1-V9 rDNA sequence (Johnson et al., 2019). And we also found Zhao and her colleagues did qPCR to measure ER-stress *via* Col1A1-expression but did not confirm P. intermedia quantities by qPCR, which is a limitation. The quantitative results of P. intermedia are always best confirmed by qPCR. At the same time, colonization of the oral microbiota depends on the balance between microbial virulence host resistance, and bacterial symbiosis (Akimbekov et al., 2022). In this regard, we suggest further determination of the virulence factors of *Prevotella*, *Porphyromonas* and other critical bacteria, as they are likewise closely related to the mechanisms of periodontitis pathogenesis and progression (Aleksijević et al., 2022).

We believe that Zhao and her colleagues have made a great contribution to the pathogenesis of periodontitis in young women with stage III periodontitis. Nonetheless, because to the numerous factors regulating the oral microbiome, researchers must perform more extensive disease screening of participants and optimize testing modalities to further clarify which bacteria play a genuinely crucial role in young women with periodontitis.

## Author contributions

WH and XT wrote the manuscript with the support from JB. The original idea was conceived by JB. All authors contributed to the article and approved the submitted version.
